# Retraction of: Gelatin-biofermentative unsulfated glycosaminoglycans semi-interpenetrating hydrogels via microbial-transglutaminase crosslinking enhance osteogenic potential of dental pulp stem cells

**DOI:** 10.1093/rb/rbag088

**Published:** 2026-05-21

This is a retraction of: Annalisa La Gatta, Virginia Tirino, Marcella Cammarota, Marcella La Noce, Antonietta Stellavato, Anna Virginia Adriana Pirozzi, Marianna Portaccio, Nadia Diano, Luigi Laino, Gianpaolo Papaccio, Chiara Schiraldi, Gelatin-biofermentative unsulfated glycosaminoglycans semi-interpenetrating hydrogels via microbial-transglutaminase crosslinking enhance osteogenic potential of dental pulp stem cells, *Regenerative Biomaterials*, Volume 8, Issue 3, June 2021, rbaa052, https://doi.org/10.1093/rb/rbaa052.

In December 2025, a reader alerted the Editors to similarities between a number of the images presented in the article and those published by the same author group in *Lasers in Medical Science* in 2020. These concerns were also raised via PubPeer (https://pubpeer.com/publications/0B53F38D11491D25C54E99A4500235).

Specifically, Figure 11A (48KDa, ACTIN at 15 and 30 days) is from Figure 4B (25KDa, AQP3) in Anna de Filippis, Antonella D’Agostino, Anna Virginia Adriana Pirozzi, Maria Antonietta Tufano, Chiara Schiraldi & Adone Baroni, Q-switched Nd-YAG laser alone and in combination with innovative hyaluronic acid gels improve keratinocytes wound healing in vitro, *Lasers in Medical Science*, Volume 36, July 2021, Pages 1047–1057, 10.1021/acs.lang muir.5b00615. The *Lasers in Medical Science* article was published online on 26 September 2020 and the *Regenerative Biomaterials* article was accepted on 15 November 2020 and published online on 12 June 2021. Furthermore, a two-gel strip (11KDa, OC at 15 and 30 days) may share the same origin within Figure 11A of this paper.

The Editors contacted the corresponding author who explained that the experiments were performed approximately six years ago and the original unprocessed image files of Figure 11A are no longer available.

The Editors investigated the concerns raised and have decided to retract this paper because the image duplication described above, and the authors’ inability to supply the raw images, undermine their confidence in the results and conclusions reported in the paper. The journal notified all authors of this decision prior to publication of this retraction notice. This decision has been made in accordance with guidance developed by the Committee on Publication Ethics (COPE).

